# Regulation of secondary cell wall biosynthesis by a NAC transcription factor from *Miscanthus*

**DOI:** 10.1002/pld3.24

**Published:** 2017-11-01

**Authors:** Philippe Golfier, Christopher Volkert, Feng He, Thomas Rausch, Sebastian Wolf

**Affiliations:** ^1^ Centre for Organismal Studies Heidelberg Department of Plant Molecular Physiology Heidelberg University Heidelberg Germany; ^2^ Centre for Organismal Studies Heidelberg Department of Cell Biology Heidelberg University Heidelberg Germany

**Keywords:** biomass, cell wall, lignin, *Miscanthus*, transcription factor, transcriptional networks

## Abstract

Cell wall recalcitrance is a major limitation for the sustainable exploitation of lignocellulosic biomass as a renewable resource. Species and hybrids of the genus *Miscanthus* have emerged as candidate crops for the production of lignocellulosic feedstock in temperate climates, and dedicated efforts are underway to improve biomass yield. However, nothing is known about the molecular players involved in *Miscanthus* cell wall biosynthesis to facilitate breeding efforts towards tailored biomass. Here, we identify a *Miscanthus sinensis* transcription factor related to SECONDARY WALL‐ASSOCIATED NAC DOMAIN1 (SND1), which acts as a master switch for the regulation of secondary cell wall formation and lignin biosynthesis. Ms *SND1* is expressed in growth stages associated with secondary cell wall formation, together with its potential targets. Consistent with this observation, Ms *SND1* was able to complement the secondary cell wall defects of the *Arabidopsis snd1 nst1* double mutant, and ectopic expression of Ms *SND1* in tobacco leaves was sufficient to trigger patterned deposition of cellulose, hemicellulose, and lignin reminiscent of xylem elements. Transgenic studies in *Arabidopsis thaliana* plants revealed that MsSND1 regulates, directly and indirectly, the expression of a broad range of genes involved in secondary cell wall formation, including MYB transcription factors which regulate only a subset of the SCW differentiation program. Together, our findings suggest that MsSND1 is a transcriptional master regulator orchestrating secondary cell wall biosynthesis in *Miscanthus*.

## 1 INTRODUCTION

The bulk of the globally available renewable biomass is formed by plant secondary cell walls (SCWs). A major limitation for tapping into the potential of this sustainable source of energy is cell wall recalcitrance, or the resistance to deconstruction, conferred by the properties of and interactions between SCW components. SCWs are deposited by specialized cells after cessation of growth and differ from the growth‐controlling primary cell wall mainly by the incorporation of the aromatic polyphenol lignin, which confers mechanical support and impregnation to water conducting tissues (Lucas et al., 2013). As lignin is extremely resistant to degradation and coats the SCW polysaccharide network of cellulose and hemicellulose, it has become the main target for efforts aiming at decreasing cell wall recalcitrance (Marriott et al., 2014; Sibout et al., 2016; Van Acker et al., 2014; Wilkerson et al., 2014). Attempts to decrease lignin content or alter lignin composition frequently caused undesirable phenotypes such as collapsed xylem vessels, dwarfing, or increased susceptibility to pathogens (Bonawitz & Chapple, 2013). However, dwarfism in response to interference with lignin biosynthesis was in some cases caused by signaling, rather than by insufficient cell wall integrity, and plants with altered lignin content, but without growth penalty could be obtained by circumventing the signaling feedback (Bonawitz et al., 2014; Gallego‐Giraldo, Escamilla‐Trevino, Jackson, & Dixon, 2011a; Gallego‐Giraldo, Jikumaru, Kamiya, Tang, & Dixon, 2011). This demonstrates that plant growth, in principle, tolerates alternative cell wall structures if secondary responses can be controlled. Thus, a thorough understanding of cell wall biosynthesis, maintenance, and perception can greatly facilitate tailoring biomass for broad application.

The herbaceous monocot genus *Miscanthus* harbors perennial C4 grasses that originate from subtropical and tropical regions in East Asia. Due to low water and modest nutrient requirements, high photosynthetic efficiency, cold and drought tolerance, and high biomass yield, *Miscanthus* has emerged as leading second‐generation bioenergy crop for the production of lignocellulosic biomass in temperate climates (Lewandowski et al., 2016; van der Weijde et al., 2013). To improve biomass composition and yield, several global breeding programs have been initiated, aiming to capitalize on existing genetic and phenotypic variation within and between *Miscanthus* species (Robson et al., 2013). In addition, these efforts are complemented by in‐depth cell wall profiling of various genotypes (da Costa et al., 2014, 2017). However, understanding of the molecular regulation involved in SCW formation in *Miscanthus* has received thus far little attention.

The biosynthesis of SCW cellulose, hemicellulose, and lignin in *Arabidopsis* is controlled by a sophisticated network of transcription factors (TFs) that coordinately integrate developmental and environmental cues into SCW formation (Taylor‐Teeples et al., 2015). The network is organized in a multitiered hierarchical manner by TFs of the NAM, ATAF1,2, and CUC2 (NAC) and MYB classes (Nakano, Yamaguchi, Endo, Rejab, & Ohtani, 2015). A group of closely related NAC TFs including NAC SECONDARY WALL THICKENING PROMOTING FACTOR1 (NST1), SECONDARY WALL‐ASSOCIATED NAC DOMAIN1 (SND1)/NST3, VASCULAR‐RELATED NAC‐DOMAIN6 (VND6), and VND7 is situated in the first tier and can be viewed as master switches regulating SCW formation (Nakano et al., 2015), which includes transcriptional upregulation of enzymes involved in SCW biosynthesis, but also controlling the machinery for its patterned deposition (Kubo et al., 2005; Oda & Fukuda, 2013). NST1 and SND1/NST3 have been shown to redundantly regulate the SCW thickening in fibers, whereas VND6 and VND7 are implicated in the SCW program of xylem elements (Kubo et al., 2005; Mitsuda, Seki, Shinozaki, & Ohme‐Takagi, 2005; Mitsuda et al., 2007; Zhong, Demura, & Ye, 2006; Zhong, Richardson, & Ye, 2007b). Downstream of NAC TFs, MYB46 and MYB83 were identified in *Arabidopsis* as second‐tier regulators of SCW formation (Ko, Kim, & Han, 2009; McCarthy, Zhong, & Ye, 2009; Zhong, Richardson, & Ye, 2007a). Targets of MYB46 and MYB83 in the third tier comprise a number of MYB TFs, such as MYB58, MYB63, and MYB85, as well as other types of TFs, which are proposed to fine‐tune transcriptional regulation of the SCW formation (Ohman et al., 2013; Zhong, Lee, Zhou, McCarthy, & Ye, 2008; Zhou, Lee, Zhong, & Ye, 2009). Finally, the actuators of the SCW differentiation program comprise biosynthetic enzymes for the main components cellulose, xylan, and monolignols, as well as enzymes involved in lignin polymerization and programmed cell death. Some of these metabolic enzymes are regulated by both the lower‐tier MYBs and the master switches, constituting feed‐forward loops (Nakano et al., 2015; Taylor‐Teeples et al., 2015). Master switches of the NAC family capable of activating the SCW differentiation program have been identified in species such as *Brachypodium*,* Eucalyptus*, maize, *Medicago*, pine, poplar, rice, and switchgrass, indicating a high degree of conservation of the regulatory network of SCW biosynthesis in vascular plants (Valdivia et al., 2013; Yoshida et al., 2013; Zhao et al., 2010; Zhong, Lee, & Ye, 2010a; Zhong et al., 2011, 2015).

Here, we identify and functionally characterize a NAC TF of the SND1 class from *Miscanthus sinensis*. Ms*SND1* is expressed in tissues undergoing SCW formation together with its potential targets. Expression of Ms*SND1* was sufficient to complement the SCW defects of the *Arabidopsis snd1 nst1* double mutant. In addition, transient expression of Ms*SND1* in tobacco leaves triggered patterned deposition of cellulose, hemicellulose, and lignin reminiscent of SCW differentiation in mesophyll cells, in sharp contrast to what was observed after expression of a lower‐tier MYB TF. Transgenic *Arabidopsis thaliana* lines carrying an inducible version of MsSND1 revealed that MsSND1 regulates directly and indirectly the expression of a broad range of genes involved in SCW formation. Together, our findings suggest that MsSND1 is a transcriptional regulator capable of orchestrating SCW biosynthesis in *Miscanthus sinensis*.

## 2 MATERIALS AND METHODS

### 2.1 Phylogeny

Amino acid sequences were aligned with ClustalW and the neighbor‐joining phylogenetic tree with 1000 bootstraps was conducted using MEGA6 software (Tamura, Stecher, Peterson, Filipski, & Kumar, 2013). References for Arabidopsis transcription factors used in the alignment are mentioned in the introduction, *Populus* sequences were taken from Ref. (Ohtani et al., 2011), *Brachypodium* sequences were taken from Ref. (Valdivia et al., 2013), maize and rice sequences were from Ref. (Zhong et al., 2011), and *Panicum* sequences were from Ref. (Zhong et al., 2015). Miscanthus sequences are listed in Table S3. Alignment of MYB transcription factors included sequences from Arabidopsis (McCarthy et al., 2009; Stracke, Werber, & Weisshaar, 2001; Zhong et al., 2006, 2007a, 2008), *Antirrhinum (Tamagnone* et al.*,* 1998 *)*,* Eucalyptus (Goicoechea* et al.*,* 2005 *; Legay* et al.*,* 2007 *)*,* Pinus (Bomal* et al.*,* 2008 *; Patzlaff* et al.*,* 2003 *)*, and *Populus* (McCarthy et al., 2010; Zhong, McCarthy, Haghighat, & Ye, 2013). Alignment of MsSND1 and AtSND1 was performed with Clustal Omega (Sievers et al., 2011).

### 2.2 Molecular cloning

Plasmid constructs were assembled via GreenGate cloning (Lampropoulos et al., 2013). The protein‐coding region of the Ms*SND1* gene was amplified by PCR using cDNA from *Miscanthus sinensis* and appropriate primers with *Bsa*I restriction site overhang (see Table S1). More details about cloning such as primers, modules, and assembled constructs for plant transformation can be found in Table S1.

### 2.3 Plant materials, growth conditions, and treatments

*Miscanthus sinensis* (identification number: Sin‐13) collected in Honshu, Japan (Clifton‐Brown & Lewandowski, 2002), were grown from seeds in glasshouse under 8/16‐hr (light/dark) photoperiod at 22–25°C. *Arabidopsis thaliana* ecotype Columbia‐0 (Col‐0) was grown in soil at 21°C under 8/16‐hr (light/dark) photoperiod until rosette stadium and then transferred to 16/8‐hr photoperiod. *Arabidopsis* plants were stably transformed by the floral dip method as described (Clough & Bent, 1998). For selection, plants were grown on half‐strength MS plates supplemented with 25 mg/L hygromycin or 7.5 mg/L glufosinate ammonium (Sigma‐Aldrich); 10‐day‐old transgenic *Arabidopsis* seedlings of mCherry‐GR‐Ms*SND1* lines were pretreated with 10 μM cycloheximide (CHX), a protein synthesis inhibitor, for 2 h. To activate mCherry‐GR‐Ms*SND1,* the seedlings were transferred to 10 μM dexamethasone (DEX) and/or 10 μM cycloheximide (CHX) solution for 4 h. *Arabidopsis snd1 nst1* double mutant seeds (Mitsuda et al., 2007) were obtained from the Nottingham *Arabidopsis* Stock Center (NASC).

### 2.4 Transient transformation of *Nicotiana benthamiana* leaves

For overexpression experiments, *Agrobacterium tumefaciens* ASE (pSOUP^+^) was transformed with the respective construct. Transgenic clones were incubated in liquid LB medium containing respective antibiotics for 2 days. The bacteria were transferred to infiltration medium (10 mM MgCl_2_, 10 mM MES, 0.15 mM acetosyringone at pH 5.7). The suspension was set to OD_600_ 0.4 and infiltrated with a needless syringe into 4‐ to 6‐week‐old *Nicotiana benthamiana* leaves. After 5 days, the leaves were embedded in 6% agarose, hand‐sectioned, and stained.

### 2.5 Tissue staining and microscopy

Lignin was stained with either HCl‐phloroglucinol or basic fuchsin as described in Ref. (Valdivia et al., 2013). Cellulose was stained with the fluorochrome calcofluor white M2R (fluorescent brightener 28). Hemicelluloses were detected with xylan and arabinoxylan‐specific LM11 as primary (McCartney, Marcus, & Knox, 2005) and Alexa 488‐conjugated donkey anti‐rat IgG (H+L; Thermo Fisher) as secondary antibody according to the procedure described elsewhere (McCartney, Steele‐King, Jordan, & Knox, 2003). Cell walls were stained with SCRI Renaissance 2200 staining (Musielak, Schenkel, Kolb, Henschen, & Bayer, 2015). HCl‐phloroglucinol staining was imaged under brightfield with a 20.0 × 0.40 NA objective on a Leica DM IRB inverted microscope. Fluorescent images were captured with a confocal Leica TCS SP5II microscope equipped with a 40.0 × 1.25 NA objective. Basic fuchsin‐ and SCRI Renaissance‐stained tissues were excited at 561 nm and 405 nm to detect emission at 593/40 nm and with DAPI filter, respectively. Calcofluor white‐stained tissues were irradiated with UV light and detected with DAPI filter. Immunolabeled tissues with LM11 and anti‐rat Alexa 488 conjugate were excited using the 488‐nm Argon laser line to detect emission with Alexa 488 filter settings. The fluorescent protein mCherry was imaged at 543‐nm excitation and 600/80‐nm detection. Orthogonal sections and scale bars were produced with ImageJ software.

### 2.6 Gene expression analysis

Developing *Miscanthus* leaves were cut at their node, dissected, and immediately frozen in liquid nitrogen. Total RNA was isolated from ground plant material from 100 mg *Arabidopsis* seedlings or 30 mg *Miscanthus* leaves using GeneMatrix Universal RNA Purification Kit (EURx/Roboklon) including an on‐column DNase I digestion. cDNA was synthesized according to the manufacturer's instructions of Superscript III Reverse Transcriptase (Invitrogen) for *Arabidopsis* samples and RevertAid First Strand cDNA Synthesis Kit (Thermo Fisher) for *Miscanthus* samples with oligo dT primer and 1 μg RNA. qPCRs were prepared in 15 μl volume containing 2 μl of 1:8 diluted cDNA, 1 μl 5 μM of each forward and reverse primer, 0.3 μl 10 mM dNTPs, 0.3 μl of 1:400 diluted SYBR^®^ Green I (Sigma‐Aldrich), 0.3 μl of JumpStart^™^ Taq DNA polymerase with 1.5 μl of the corresponding buffer (Sigma‐Aldrich), and 8.6 μl H_2_O. Ct values, primer efficiencies, and melting curves were measured in a Rotor‐Gene Q thermocycler and evaluated by Q Series Software Q (Qiagen). Primer sequences can be found in Table S2. Stably expressed housekeeping genes in *Miscanthus sinensis* leaf gradient were identified in a *Miscanthus* transcriptome (Barling et al., 2013) by blasting common housekeeping genes from *Arabidopsis* (Czechowski, Stitt, Altmann, Udvardi, & Scheible, 2005). Primers were designed for *protein phosphatase 2A subunit 3A* (*PP2A*), clathrin adaptor *AP2M* (*Clath*), *peroxin 4* (*UBC21*), and *TIP41‐like protein* (*TIP*) based on sequences in the *Miscanthus* transcriptome (Table S2). During the course of the leaf gradient, cytoskeleton reference genes like tubulin or actin were differentially expressed in leaf base and tip. Hence, the most stable reference genes *PP2A* and *UBC21* were selected for normalization. Geometrical mean of three biological replicates is shown. Gene expression of *Arabidopsis* was normalized against clathrin adaptor subunit (At5g46630) described in (Czechowski et al., 2005). The qPCR products were either sequenced or checked on acrylamide gel in cases of small amplicons.

### 2.7 Accession numbers

Sequence data from this article can be found in the GenBank databases under the following accession numbers: KY930620 for MsSND1; KY930621 for MsSCM1; MF996502 for MsSCM2; KY930622 for MsSCM3; MF996501 for MsSCM4; and KY930623 for MsLAC2. Arabidopsis accession numbers are as follows: At1 g32770 for AtSND1; At4g35350 for XCP1; At5g12870 for MYB46; At3g08500 for MYB83; At5g44030 for CesA4; At5g17420 for CesA7; At4g18780 for CesA8; At2g38080 for LAC4; At2g28110 for IRX7; At5g54690 for *IRX8*; At1g51680 for *4CL1*; At4g34050 for *CCoAOMT1*; At5g46630 for *Clathrin*; At5g25760 for *UBC21*; and At1g13320 for *PP2A*.

## 3 RESULTS AND DISCUSSION

### 3.1 Identification of *Miscanthus sinensis SND1* through phylogenetic analysis of the NAC transcription factor family

**Figure 1:**
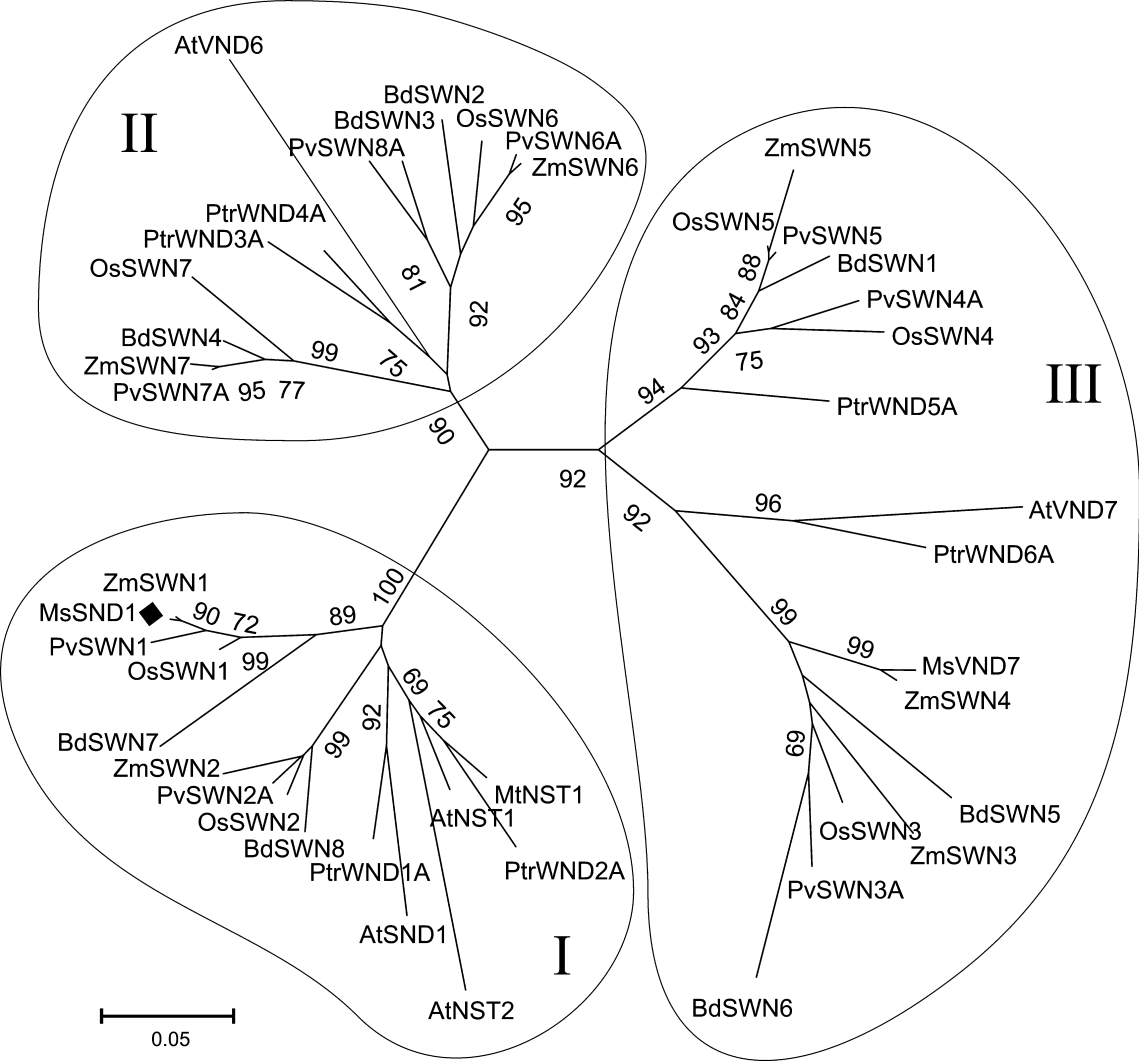
Identification of Ms *SND1*. Phylogeny of MsSND1 in relation to the NAC transcription factor subfamily Ic from angiosperm lineages *Arabidopsis*,* Brachypodium*,* Panicum*,* Populus*,* Oryza,* and *Zea*. Amino acid sequences were aligned with ClustalW, and the neighbor‐joining phylogenetic tree with 1000 bootstraps was conducted using MEGA6 software (Tamura et al., 2013)

In *Miscanthus*, the molecular players involved in cell wall biosynthesis are largely unknown. To facilitate the identification of potential targets for future crop improvement, we queried the previously published *Miscanthus* transcriptome (Barling et al., 2013) for TFs involved in SCW biosynthesis using known factors from *Arabidopsis thaliana* and other plants. A phylogenetic analysis of NAC TFs from different angiosperm lineages showed that the subfamily Ic may be further divided into three classes (Figure 1). Members of classes II and III are implicated in xylem differentiation (Kubo et al., 2005; Zhou, Zhong, & Ye, 2014), whereas members of class I are described to be involved in fiber differentiation (Zhong et al., 2006). In the *Miscanthus* transcriptome, we found various transcripts putatively encoding SCW TFs and concentrated on the *Miscanthus sinensis* SND1 candidate. The predicted protein shares 41.5% identity and 51.5% similarity on amino acid level with AtSND1 (Fig. S1). The structurally and functionally essential N‐terminal DNA‐binding motif, known as the NAC domain, shows the highest similarity with 93% within the amino acid sequence, suggesting that MsSND1 may possess similar DNA‐binding characteristics as AtSND1. The more divergent C‐terminus that contains the transcriptional activation domain (Duval, Hsieh, Kim, & Thomas, 2002; Zhong et al., 2006) is 63 amino acids longer in MsSND1 compared to AtSND1 that may reflect different activation properties. In summary, our analysis indicates a close relationship between MsSND1 and AtSND1.

### 3.2 High Ms*SND1* expression coincides with vascular development and SCW formation in *Miscanthus* leaves

**Figure 2:**
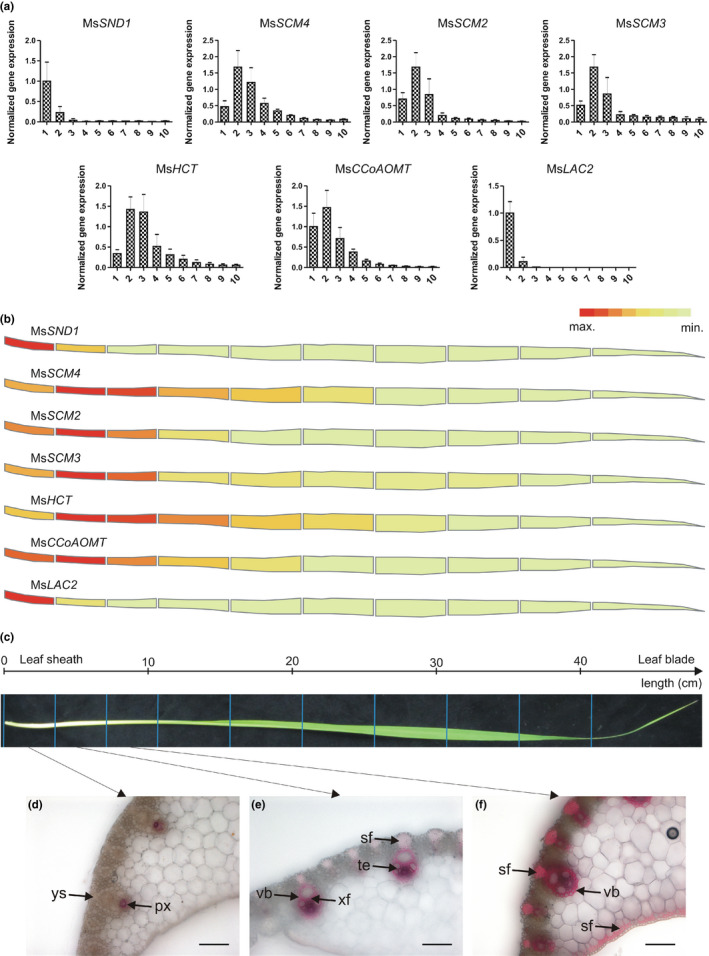
Ms *SND1* expression is correlated with vascular development and with the expression of its putative target genes *Miscanthus sinensis* leaves. (a,b) Gene expression profiles of the indicated genes over ten developmental zones as obtained by quantitative real‐time PCR. The results of three biological replicates were combined, normalized against two reference genes (PP2A and UBC, [a]) and visualized as heat map in (b). Pictures of all three leaves are shown in Fig. S1. Cross‐sections of the first three basal zones (d–f) from the *Miscanthus* leaf depicted in (c) were stained for lignin with HCl‐phloroglucinol, indicating that the expression of Ms *SND1* and its putative targets is concomitant with the onset of vascular development. Scale bars: 100 μm. px, protoxylem; sf, sclerenchyma fibers (extraxylary fibers); te, tracheary elements; vb, vascular bundle; xf, xylary fibers; ys, young sclerenchyma

In monocots, leaf differentiation follows a linear pattern from the leaf sheath toward the leaf blade, resulting in a continuous developmental gradient along the leaf. To explore the role of Ms*SND1* in *Miscanthus* development, the expression profile of Ms*SND1* together with those of putative cell wall‐related transcription factors and biosynthetic genes was determined along the developmental gradient in the leaf and visualized (Figure 2a,b, Fig. S2). The transcripts of R2R3‐MYB TFs, lignin biosynthesis, and polymerization genes were identified in the *Miscanthus* transcriptome in the same manner as described for Ms*SND1*. Along the leaf developmental gradient, the expression of Ms*SND1* was highest at the leaf sheath and decreased rapidly over the following two leaf segments (Figure 2a,b). We then analyzed, the expression pattern of putative downstream targets of SND1 related to AtMYBs involved in the SCW transcriptional network. In this case, relationship to previously described factors was more ambiguous; therefore, we named them *Miscanthus sinensis* SECONDARY CELL WALL MYBs 1‐4 (MsSCM1‐4). MsSCM1‐3 fall into the clade of AtMYB20, AtMYB43, and AtMYB85, respectively, whereas MsSCM4 is most closely related to AtMYB63 and AtMYB58 (Fig. S3) (Zhong et al., 2008; Zhou et al., 2009). The expression of these MYB TFs, as well as that of lignin biosynthesis genes peaked in the second segment to decline toward the leaf blade at different rates depending on the gene. Putative orthologues from maize and rice revealed very similar expression patterns, supporting our results (Li et al., 2010; Wang et al., 2014). These observations suggest evolutionary conservation of the SCW transcriptional network in accordance with previous reports (Zhong et al., 2010a), and demonstrate feasibility of a homology‐based approach for identifying SCW‐related genes in *Miscanthus*.

To corroborate these results, the expression profiles of the putative SCW‐related genes were correlated with SCW differentiation in leaf segments of *Miscanthus* (Figure 2d–f). At the leaf sheath, the fundamental organization of vasculature is initiated very early in development. Lignification of vascular tissue started in protoxylem cells of the first segment, shortly followed by lignification of tracheary elements and sclerenchyma fibers (extraxylary fibers) in the second and third leaf segment (Figure 2d–f). SCW deposition and lignification of cells within the vascular bundles appeared more prominent than in sclerenchyma fibers (Figure 2e), which may be required to secure integrity of the tissue during early stages of leaf maturation. Extraxylary fibers, the adaxial threads of sclerenchyma fibers that are in contact with the outer bundle sheath, and the thin abaxial subepidermal strip of sclerenchyma fibers seemed to lignify and deposit SCWs in a similar fashion, different from vascular cells. This was further confirmed by basic fuchsin staining of the leaf cross‐sections. Basic fuchsin was able to stain cells in the vascular tissue but was unable to stain sclerenchyma fibers (Fig. S2b–e). Notably, sclerenchyma cells were found to possess a distinct lignin composition in alfalfa (Vallet, Chabbert, Czaninski, & Monties, 1996), which is probably the result of different lignification mechanisms employed by xylary and extraxylary fibers (Smith et al., 2013). The high expression of Ms*SND1*, candidate MYB TFs, and lignin biosynthesis genes that occurs concurrently with differentiation and SCW formation of xylary and extraxylary elements suggests a possible involvement of the investigated genes in this process.

### 3.3 Ms*SND1* complements the SCW deficiency of the *Arabidopsis snd1 nst1* double mutant

**Figure 3:**
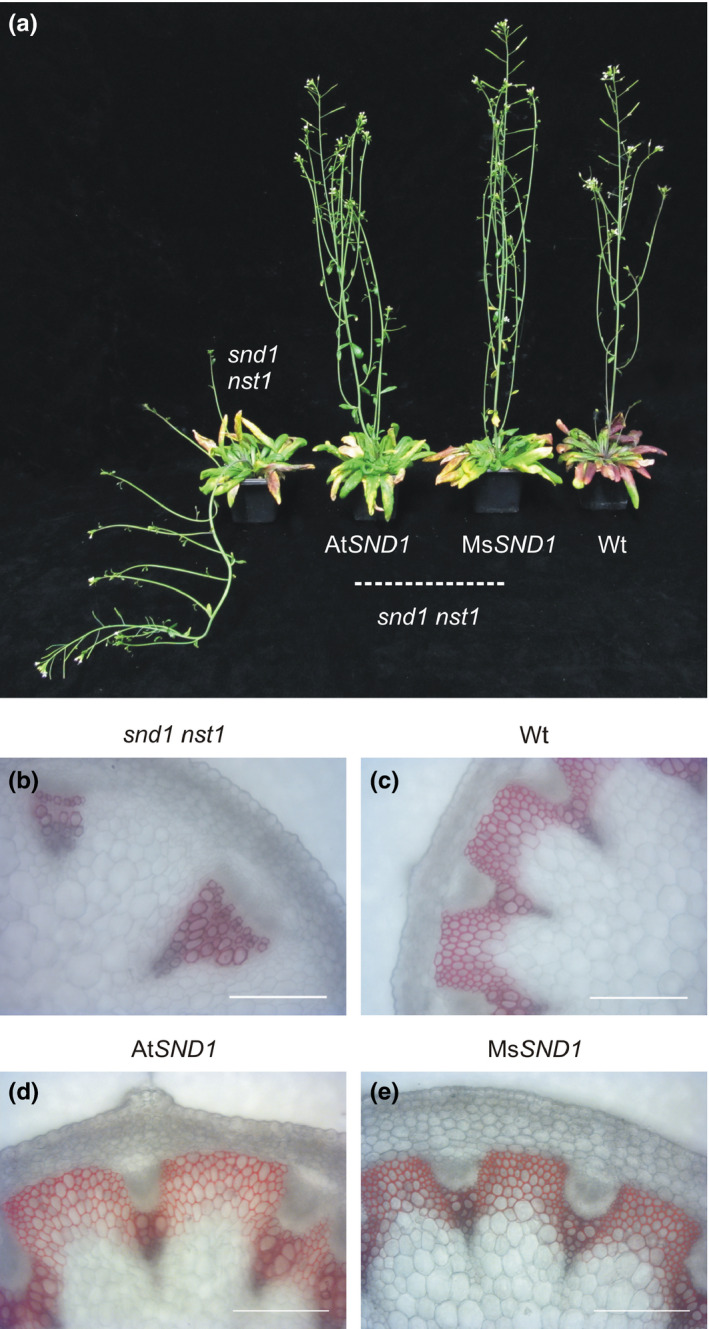
Ms *SND1* can replace Arabidopsis *SND1*. Complementation of *Arabidopsis snd1 nst1* double mutant by expression of Ms *SND1* under control of the At *SND1* promoter. Ms *SND1* restored vertical growth (a) and lignification of secondary cell walls in the interfascicular fibers similar to the positive control At *SND1* (b–e). Cross‐sections of inflorescence stems were stained with HCl‐phloroglucinol for lignin. Scale bars: 100 μm

To substantiate the hypothesis that MsSND1 is functionally equivalent to AtSND1*,* we tested whether expression under control of the At*SND1* promoter could complement the pendant stem phenotype caused by SCW defects of interfascicular fibers in the *Arabidopsis snd1 nst1* double mutant (Mitsuda et al., 2007; Figure 3a). MsSND1 was capable to rescue the vertical growth of *snd1 nst1* similar to AtSND1 (Figure 3a). Cross‐sections of inflorescence stems stained for lignin confirmed that both Ms*SND1* and At*SND1* are able to complement the *snd1 nst1* double mutant and restore loss of lignin in stem fiber cells (Figure 3d). Even though this result supported our hypothesis that MsSND1 and AtSND1 are functionally similar, a previous study has revealed that only partial activation of the regulatory network is already sufficient to rescue the *snd1 nst1* mutant, albeit with compositional changes to the SCW (Sakamoto & Mitsuda, 2015). Therefore, we continued with a more profound functional characterization of MsSND1 to shed light on its regulatory role in SCW formation.

### 3.4 Expression of Ms*SND1* leads to strong ectopic SCW deposition in tobacco leaves

**Figure 4:**
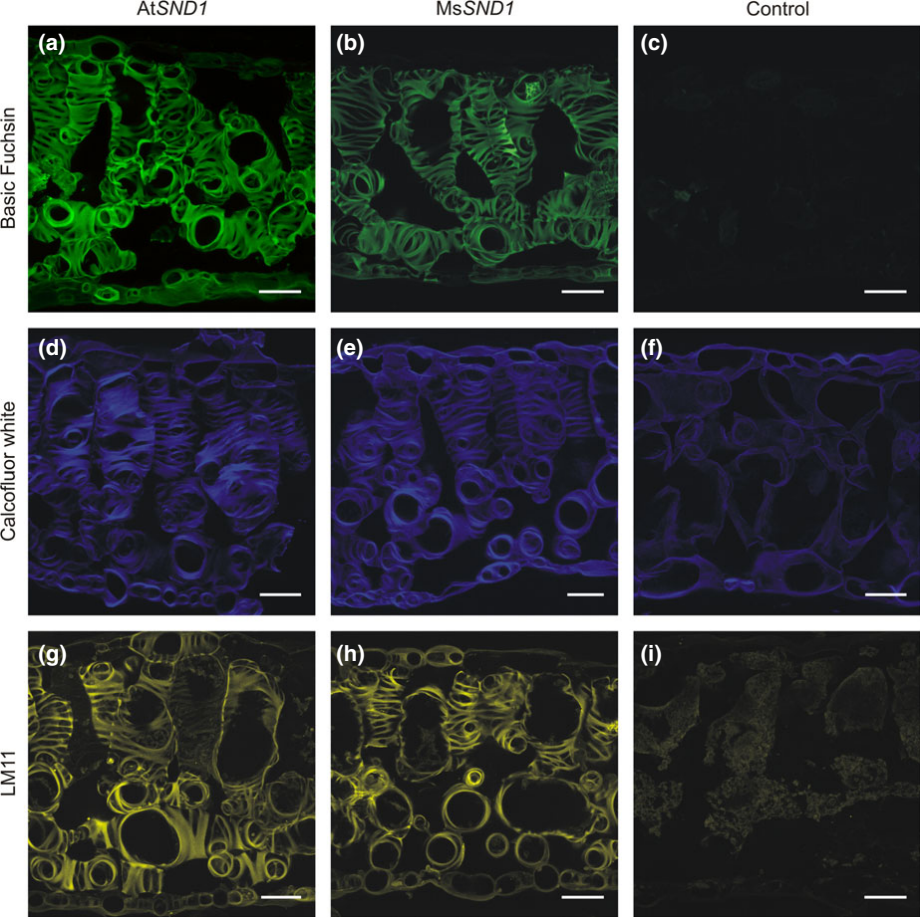
Ectopic expression of MsSND1 is sufficient to induce secondary cell wall deposition and patterning. Ectopic deposition of secondary cell wall components in *Nicotiana benthamiana* leaves induced by transient expression of At *SND1* and Ms *SND1*. Cross‐sections of tobacco leaves stained for lignin with basic fuchsin (a–c), for cellulose with Calcofluor White (d–f) and for xylan and arabinoxylan with LM11 as primary and Alexa 488 as secondary antibody (g–i). The control leaves were transformed with an empty vector control (c, f, i). Scale bar 40 μm

**Figure 5:**
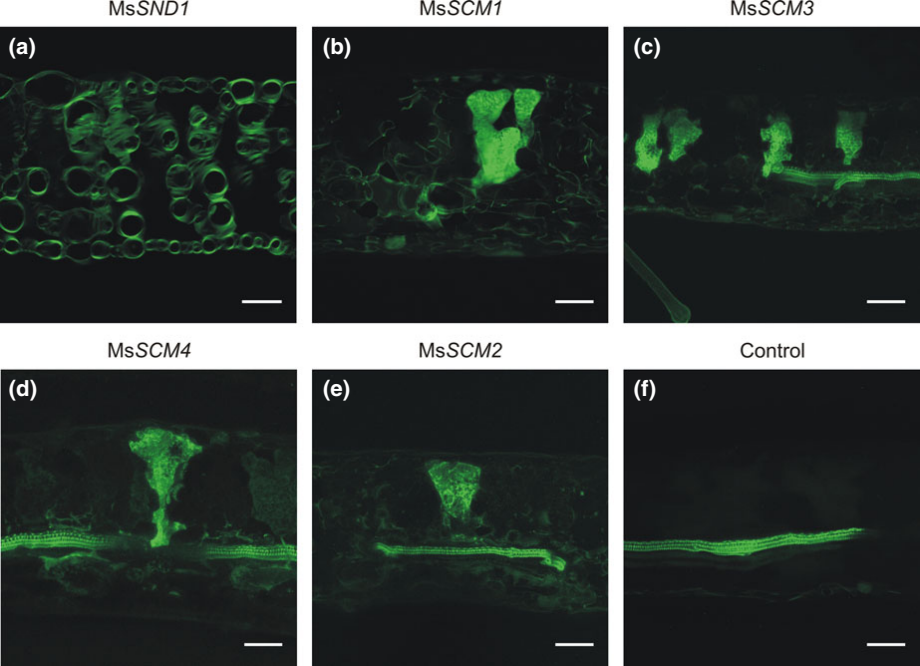
Ectopic secondary cell wall patterning differs between MsSND1 and downstream MYB factors. Ectopic deposition of lignin in tobacco leaves induced by ectopic, transient expression of Ms *SND1*, MsSCM1, MsSCM3, MsSCM4, and MsSCM2 in *Nicotiana benthamiana* leaves. Cross‐sections of leaves were stained for lignin with basic fuchsin. The control leaves were transformed with an empty vector control. Scale bar: 50 μm

In order to examine the ability of Ms*SND1* to stimulate SCW formation, we transiently overexpressed Ms*SND1* in *Nicotiana benthamiana* leaves. After 6 days of incubation, leaf cross‐sections were stained for lignin, cellulose, and hemicellulose. Overexpression of Ms*SND1* and At*SND1* led to patterned ectopic deposition of lignified SCW by epidermis, palisade cells, and spongy mesophyll cells (Figure 4). Interestingly, the patterns of ectopic SCW deposition are reminiscent of tracheary elements which has been previously observed after induced overexpression of NAC and MYB SCW master switches from several species such as Arabidopsis, poplar, *Eucalyptus*, switchgrass, rice, maize, and *Brachypodium* (Kubo et al., 2005; Zhong et al., 2007a,b; Zhong et al., 2010a; Zhong, Lee, & Ye, 2010b; Zhong et al., 2011; McCarthy et al., 2009; Valdivia et al., 2013; Yoshida et al., 2013). Cortical microtubules have long been known to play a crucial role in patterned deposition of SCW by guiding exocyst complex and secretory vesicles at the sites of SCW deposition (Baskin, 2001; Oda & Fukuda, 2013; Vukasinovic et al., 2017; Watanabe et al., 2015; Wightman & Turner, 2008). Indeed, several genes involved in cytoskeleton organization and vesicle transport like MIDD1, FRA1, kinesins, or tubulin were found to be regulated by NAC TFs (Ohashi‐Ito, Oda, & Fukuda, 2010; Yamaguchi et al., 2011; Zhong et al., 2010b). Therefore, it is tempting to speculate that overexpression of Ms*SND1* does not only activate the biosynthetic cell wall machinery but also impacts cytoskeleton organization and vesicle transport resulting in the specific patterns of SCW deposition. The high similarity of the observed SCW patterns and deposition after overexpression of At*SND1* and Ms*SND1* supports the assumption that they are functionally similar. In sharp contrast, expression of MsSCM1‐3 (related to AtMYB20, AtMYB43, and AtMYB85) and MsSCM4 (related to AtMYB63 and AtMYB58) (Fig. S3) resulted in uniform lignin deposition around *N. benthamiana* mesophyll cells without apparent patterning (Figure 5), demonstrating that individual SCW TFs differ in their functionality and target spectrum. Taken together, transient expression of Ms*SND1* in tobacco leaves demonstrates that Ms*SND1* is capable of initiating a SCW differentiation program resulting in ectopic deposition of patterned cellulose, hemicellulose, and lignin.

### 3.5 Induction of mCherry‐GR‐MsSND1 leads to transdifferentiation of xylem‐like elements and allows localization and tracking of the fusion protein

**Figure 6:**
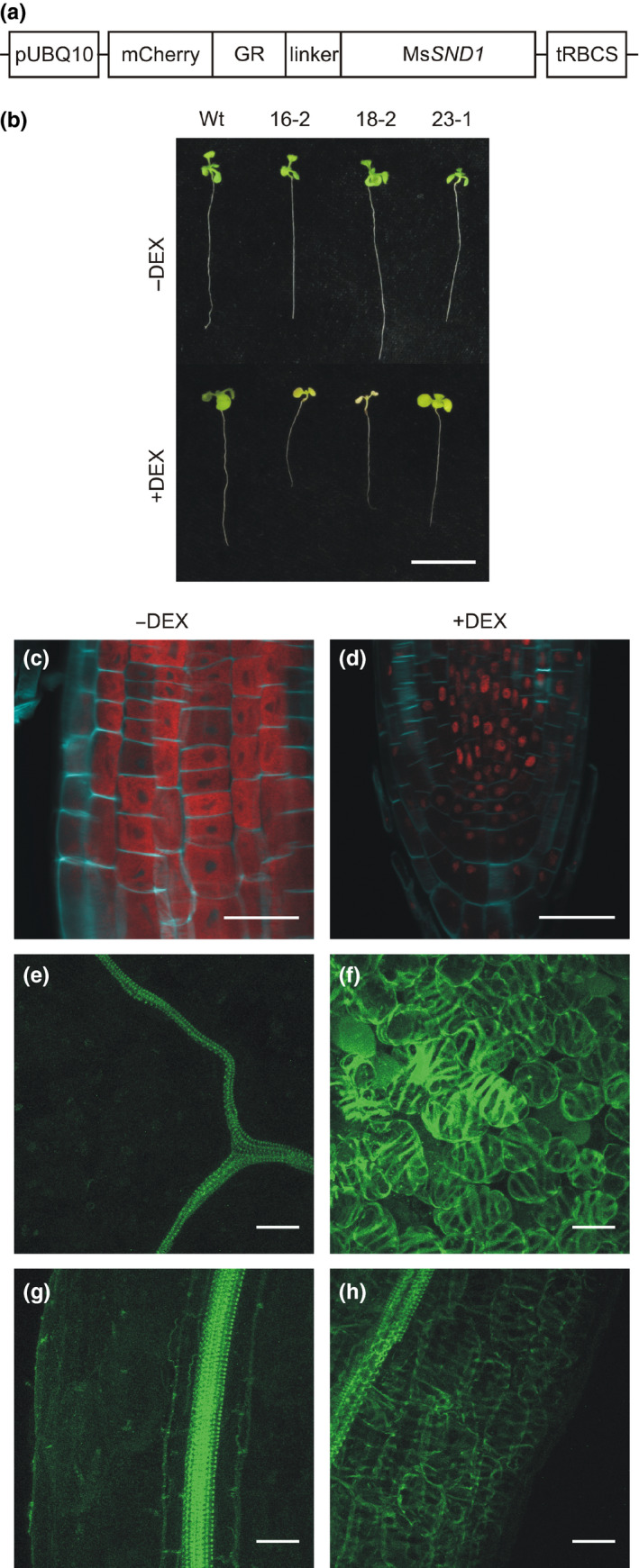
Induction of MsSND2 leads to secondary cell wall differentiation in Arabidopsis. (a) Schematic of inducible mCherry‐GR‐Ms *SND1* construct. Transgenic *Arabidopsis* mCherry‐GR‐Ms *SND1* seedlings (line 16‐2) were treated with (+DEX) and without (‐DEX) dexamethasone for 7 days (b), 4 days (e–h), and 24 hr (c, d), respectively. (c, d) The root tip cell walls were stained with SCRI Renaissance 2200, and lignin was stained with basic fuchsin. pUBQ10, ubiquitin‐10 promoter; mCherry, synthetic fluorophore; GR, ligand‐binding domain of the rat glucocorticoid receptor; tRBCS, ribulose‐1,5‐bisphosphate carboxylase small subunit terminator (from pea). Scale bar 1 cm (b) and 30 μm (c–h), respectively

To further evaluate the function of Ms*SND1*, we established a posttranslational MsSND1 induction system in *Arabidopsis* consisting of MsSND1 fused to the fluorophore mCherry and the ligand‐binding domain of the rat glucocorticoid receptor (GR; Figure 6a). Briefly, in the absence of the steroid ligand, the heat‐shock protein 90 (HSP90) builds a cytosolic complex with GR, preventing the fusion protein, in this case mCherry‐GR‐MsSND1 from entering the nucleus (Dalman, Scherrer, Taylor, Akil, & Pratt, 1991). Upon application of the synthetic steroid dexamethasone (DEX), mCherry‐GR‐MsSND1 is released from cytosolic retention and able to act as transcriptional regulator in the nucleus. Under normal growth conditions, transgenic *Arabidopsis* carrying a constitutively expressed mCherry‐GR‐Ms*SND1* construct exhibited no obvious differences to wild‐type plants concerning germination, growth, or development (Figure 6b). In contrast, activation of mCherry‐GR‐MsSND1 with DEX for 7 days led to an arrest of growth in seedlings of three independent transgenic *Arabidopsis* lines (Figure 6b). Furthermore, induction of mCherry‐GR‐MsSND1 resulted in cell death, as indicated by loss of chlorophyll. The transgenic line 16‐2 seemed to show the strongest activation of mCherry‐GR‐MsSND1, as growth was immediately arrested and loss of chlorophyll was the fastest, and thus was chosen for subsequent experiments. Nonetheless, cell death after mCherry‐GR‐MsSND1 activation occurred much slower than observed in mCherry‐GR‐Ms*VND7* lines (Fig. S4) and previously described (Yamaguchi et al., 2010), suggesting functional similarity to class I NAC TFs like At*SND1* or Bd*SWN8*, as they share a weaker induction of cell death (Valdivia et al., 2013).

In untreated mCherry‐GR‐Ms*SND1* seedlings, detection of the mCherry signal in root tips revealed a cytosolic localization of the fusion protein mCherry‐GR‐MsSND1 (Figure 6c). After 24 h of DEX treatment, the mCherry signal was exclusively detected in nuclei of root cells (Figure 6d). The sharp boundaries of the mCherry signal, excluding nuclei, in Figure 6c indicate that the mCherry‐GR‐MsSND1 fusion protein only marginally leaked into the nucleus, consistent with normal growth and development of noninduced seedlings. This was further validated by lignin staining using basic fuchsin in the absence and presence of DEX. In noninduced seedlings, lignification was only detected in vascular elements (Figure 6e, g). In contrast, DEX application for 4 days led to ubiquitous SCW deposition in the seedling including leaf mesophyll cells and root ground tissue (Figure 6f, h). In line with what was observed after transient expression of Ms*SND1* in *Nicotiana benthamiana* leaves, induction of mCherry‐GR‐MsSND1 in *Arabidopsis* gave rise to patterns of SCW deposition reminiscent of tracheary elements. This result again supports a high degree of conservation of the molecular mechanism involved in SCW patterning and biosynthesis throughout vascular plants (Bomal et al., 2008; Li et al., 2012; Zhong et al., 2010a). The establishment of a precisely inducible mCherry‐GR‐MsSND1 *Arabidopsis* line allows localization and tracking of the fusion protein and corroborates the finding that MsSND1 is capable of activating the SCW program resulting in patterned SCW deposition reminiscent of xylem elements.

### 3.6 Induction of mCherry‐GR‐MsSND1 activates expression of genes involved in SCW formation

**Figure 7:**
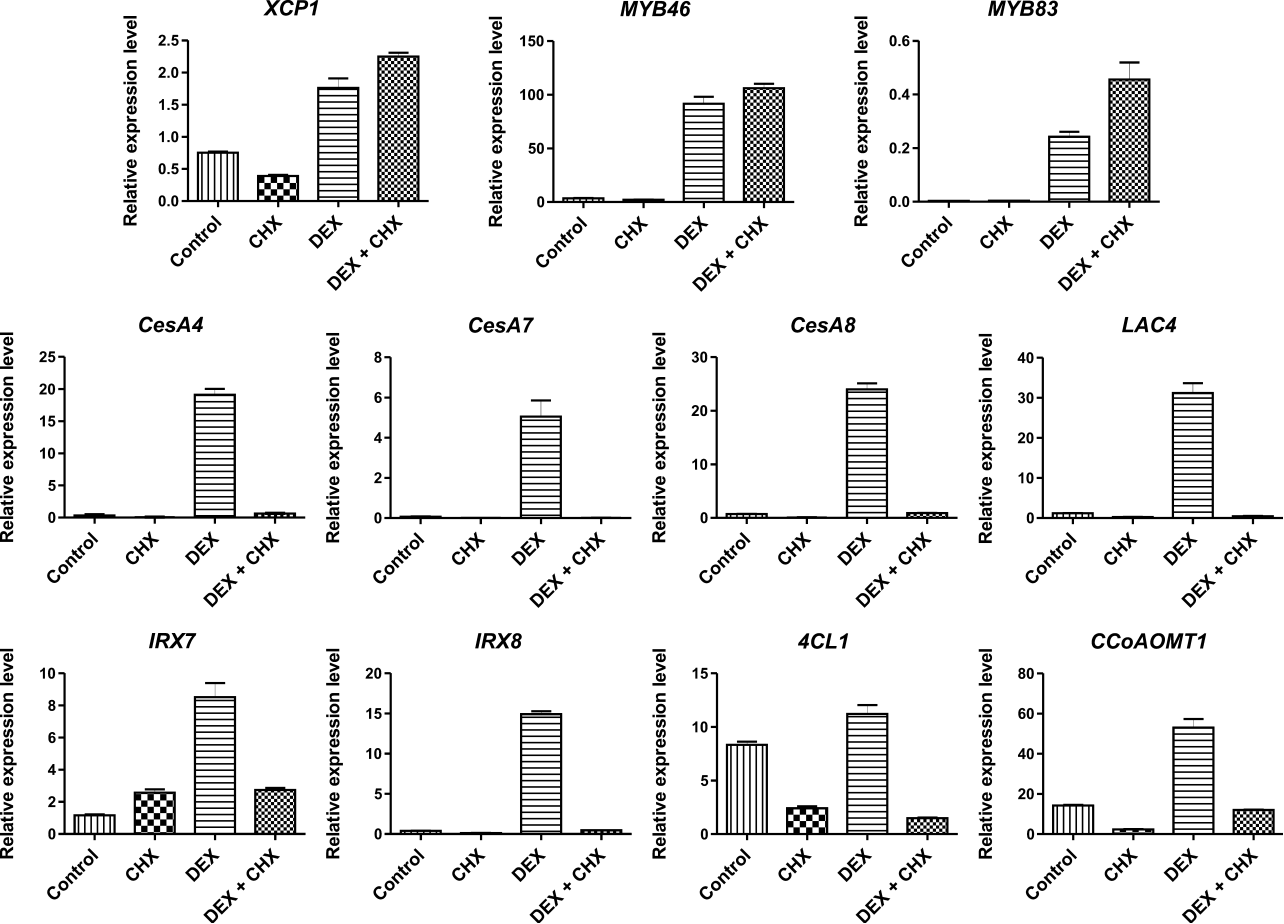
Induction of mCherry‐GR‐MsSND1 activates expression of genes involved in SCW formation. Expression analysis of candidate genes directly and indirectly targeted by MsSND1. Ten‐day‐old heterozygous *Arabidopsis* mCherry‐GR‐Ms *SND1* seedlings (line 16) were treated with cycloheximide (CHX) and/or dexamethasone (DEX). Gene expression is normalized against clathrin adaptor subunit. Bars depict means ± *SE* from three technical replicates. The same experiment was performed with an independent transgenic line with similar results (Fig. S5)

Next, we assessed the ability of MsSND1 to act as transcriptional regulator of SCW formation. Therefore, we induced mCherry‐GR‐Ms*SND1 Arabidopsis* lines with or without pretreatment with the protein translation inhibitor cycloheximide (CHX), to determine direct and indirect targets. Expression levels of selected genes involved in SCW formation were measured via qRT‐PCR (Figure 7). Activation of mCherry‐GR‐MsSND1 with DEX elevated the transcript levels of genes associated with cellulose biosynthesis (*CesA4*,* CesA7,* and *CesA8*), xylan biosynthesis (*IRX7* and *IRX8*), lignin biosynthesis (*4CL1* and *CCoAOMT1*), cell death (*XCP1*), lignin polymerization (*LAC4*), and MYB TFs (*MYB46/83*). Besides for *XCP1*,* 4Cl1,* and *CCoAOMT1,* the background expression levels in the controls were very low. Upon DEX application, gene expression was strongly upregulated up to 70‐fold in case of *CesA7* (Figure 7). However, induction of mCherry‐GR‐MsSND1 with DEX in the presence of CHX promoted the expression of *XCP*,* MYB46*, and *MYB83*, while genes associated with cellulose, hemicellulose, lignin biosynthesis, and polymerization seem to be indirect targets. The independent mCherry‐GR‐Ms*SND1* line 18 exhibited the same activation pattern as mCherry‐GR‐Ms*SND1* line 16 but with generally lower relative values (Fig. S5). Thus, MsSND1 can activate many of the same downstream target genes as previously identified for secondary wall NACs (SWNs) like AtSND1, AtVND6, and AtVND7 (Ohashi‐Ito et al., 2010; Zhong et al., 2010b).

## 4 CONCLUSION

Cell wall recalcitrance, which is mainly conferred by incorporation of lignin into SCWs, remains a major limitation to explore lignocellulosic biomass as renewable resource. Concomitantly, considerable efforts are directed at increasing production of lignocellulosic biomass that can be valorized in a sustainable manner. However, the molecular mechanisms involved in lignification and SCW formation in *Miscanthus* have received only little attention. In this study, we identified and functionally characterized *Miscanthus sinensis* SND1 as a transcriptional master regulator orchestrating SCW formation, most likely in extraxylary fibers. In addition, we could identify other TF from the SCW transcriptional network, which appears to only regulate a subset of the differentiation program. Despite the fact that research on *Miscanthus* is still hampered by challenging transformation procedures, our approach shows that utilization of the increasingly available genomic resources and transfer of knowledge from model plants greatly facilitate the investigation of molecular mechanisms. The functional characterization of MsSND1 contributes to a deeper understanding of the molecular network of TFs involved in SCW formation in *Miscanthus*. It is tempting to speculate that lower‐tier TFs such as MsSCM4 (related to AtMYB63/AtMYB58) could affect lignin qualities more specifically, suggesting that may serve as potential targets for breeding programs. Moreover, the possible involvement of Ms*SND1* in extraxylary fibers differentiation may represent a target for cell wall engineering (Yang et al., 2013) as it offers the opportunity to manipulate lignin specifically in extraxylary fibers without affecting xylem elements and thereby bypassing severe growth defects.

## CONFLICT OF INTEREST

The authors declare no conflict of interest.

## AUTHOR CONTRIBUTIONS

P.G., T.R., and S.W. designed research; P.G., C.V., and F.H. performed research and analyzed data; P.G., T.R., and S.W. wrote the manuscript.
