# Supplementary material for: Regulation of secondary cell wall biosynthesis by a NAC transcription factor from Miscanthus
Source: Plant Direct. 2017 Nov 1;1(5):e00024. doi: 10.1002/pld3.24 (PMC6508536; doi:10.1002/pld3.24)
Supplement: Supplementary file 1 [file PLD3-1-e00024-s001.pdf]

Supplemental Material

# Regulation of secondary cell wall biosynthesis by a NAC transcription factor from Miscanthus

**Philippe Golfier[1], Christopher Volkert[1], Feng He[1], Thomas Rausch[1,*], Sebastian Wolf [2,*]**

```
MsSND1      -----MSISVNGQSVVPPGFRFHPTEEELLTYYLKKKVASERIDLDVIRDVDLNKLEPWD
AtSND1      MADNKVNLSINGQSKVPPGFRFHPTEEELLHYYLRKKVNSQKIDLDVIREVDLNKLEPWD
                 :.:*:**** *************** ***:*** *::*******:**********

MsSND1      IQEKCRIGSGPQNDWYFFSHKDKKYPTGTRTNRATAAGFWKATGRDKAIYASGARRIGMR
AtSND1      IQEECRIGSTPQNDWYFFSHKDKKYPTGTRTNRATVAGFWKATGRDKII-CSCVRRIGLR
            ***:***** *************************.*********** * .* .****:*

MsSND1      KTLVFYKGRAPHGQKSDWIMHEYRLEAALDAAAGSAAHHPAAGAAADHPYYTSSPPALPT
AtSND1      KTLVFYKGRAPHGQKSDWIMHEYRLDDTPMSNGYA-------------------------
            *************************: :  : . :

MsSND1      AIRGAAAEQAAQEQEGWVICRVFKKKNLVHHGQSSGAGVTAAGNHAASKMAAAAAPMDSS
AtSND1      --DVVTEDPMSYNEEGWVVCRVFRKKNYQKIDDCPKITLSSL-------------PDDT-
                .: :  : ::****:****:***  : .:.    :::              * *:

MsSND1      PSHCSSVTVSDYSNKQQAQAMLQHSASDDALDHILQYMGGGGKQPDTKPALLDHHHHHVA
AtSND1      --------------EEEKGPTFHNTQNVTGLDHVLLYMDRTGSNICMPESQTTTQHQ---
                          :::    :::: .  .***:* **.  *.:     :    :*:

MsSND1      AATTTTAACPAGVGGLYGKFMKLPPLEHAGACGLL---PSPPGACE-----YGAADASEI
AtSND1      ---------------DDVLFMQLPSLETPKSESPVDQSFLTPSKLDFSPVQEKITERPVC
                               **:** **   : . :      *.  :        ::

MsSND1      ADWDALDRLAAYELNGLSDASKNMSAFFDVEHASAAAAFSSSSSAHVSAAVDGDLWSLAR
AtSND1      SNWASLDRLVAWQLNNGHHNPCHRKSFDEEEEN-GDT-MMQRWDLHWNNDDNVDLWSSFT
            ::* :****.*::**.  .   : .:* : *.  . : : .  . * .   : ****

MsSND1      SVS-ALHADLTMNNF
AtSND1      ESSSSLDPLLHLSV-
            . * :*.  * :.
```

**Supplemental Figure 1.** Clustal Omega (Sievers et al., 2011) alignment of *Ms*SND1 and *At*SND1. The NAC transcription factor domain is highlighted in grey.

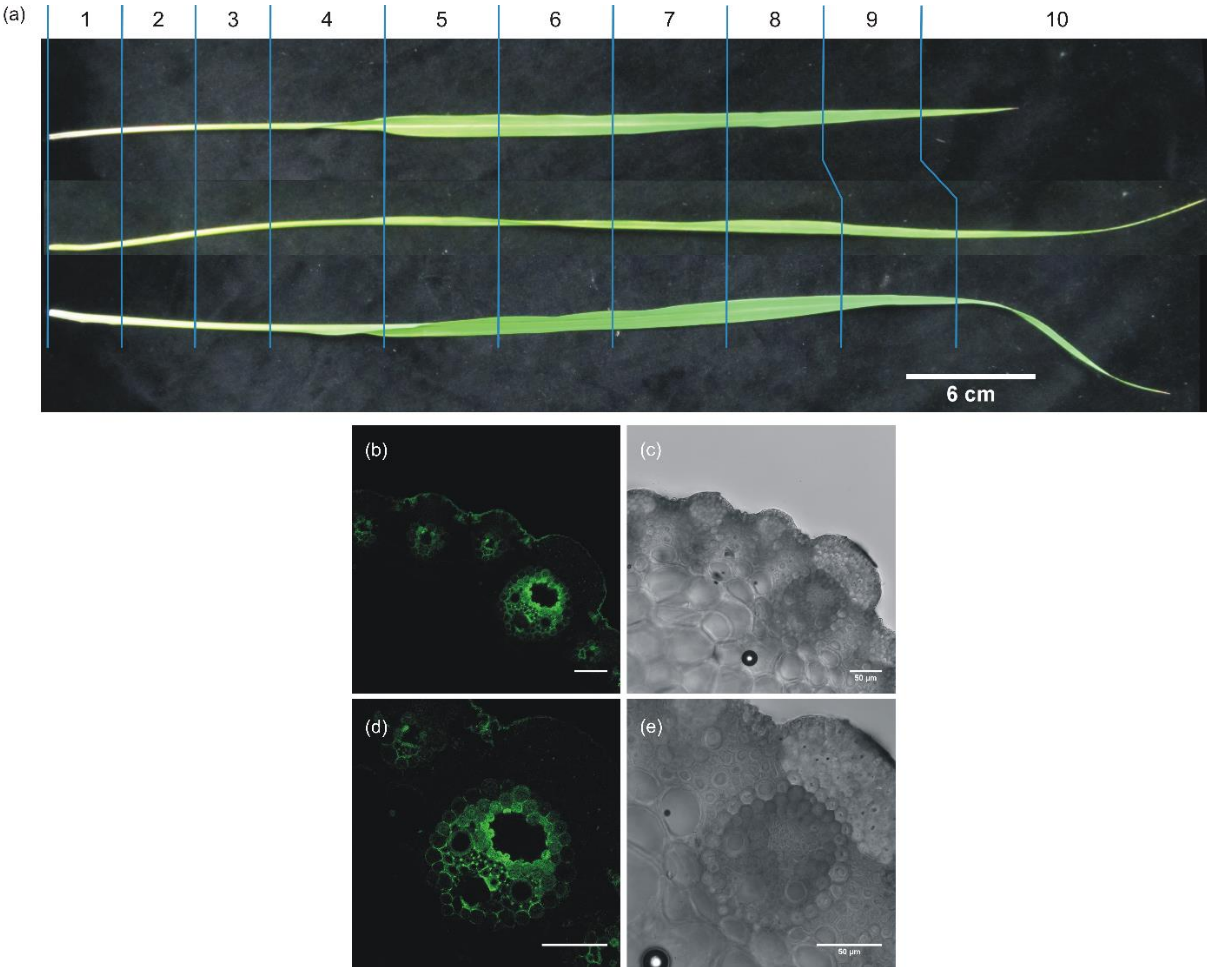


**Supplemental Figure 2.** Sampling scheme of Miscanthus leaves for expression profile analysis along the leaf gradient and cross-section of Miscanthus leaf. (A) indication of leaf sections harvested for expression analysis depicted in Fig. 2. (B-E) Cross sections through a Miscanthus leaf stained with basic fuchsin. Fluorescence (B, D) and white light images (C, E) are depicted.

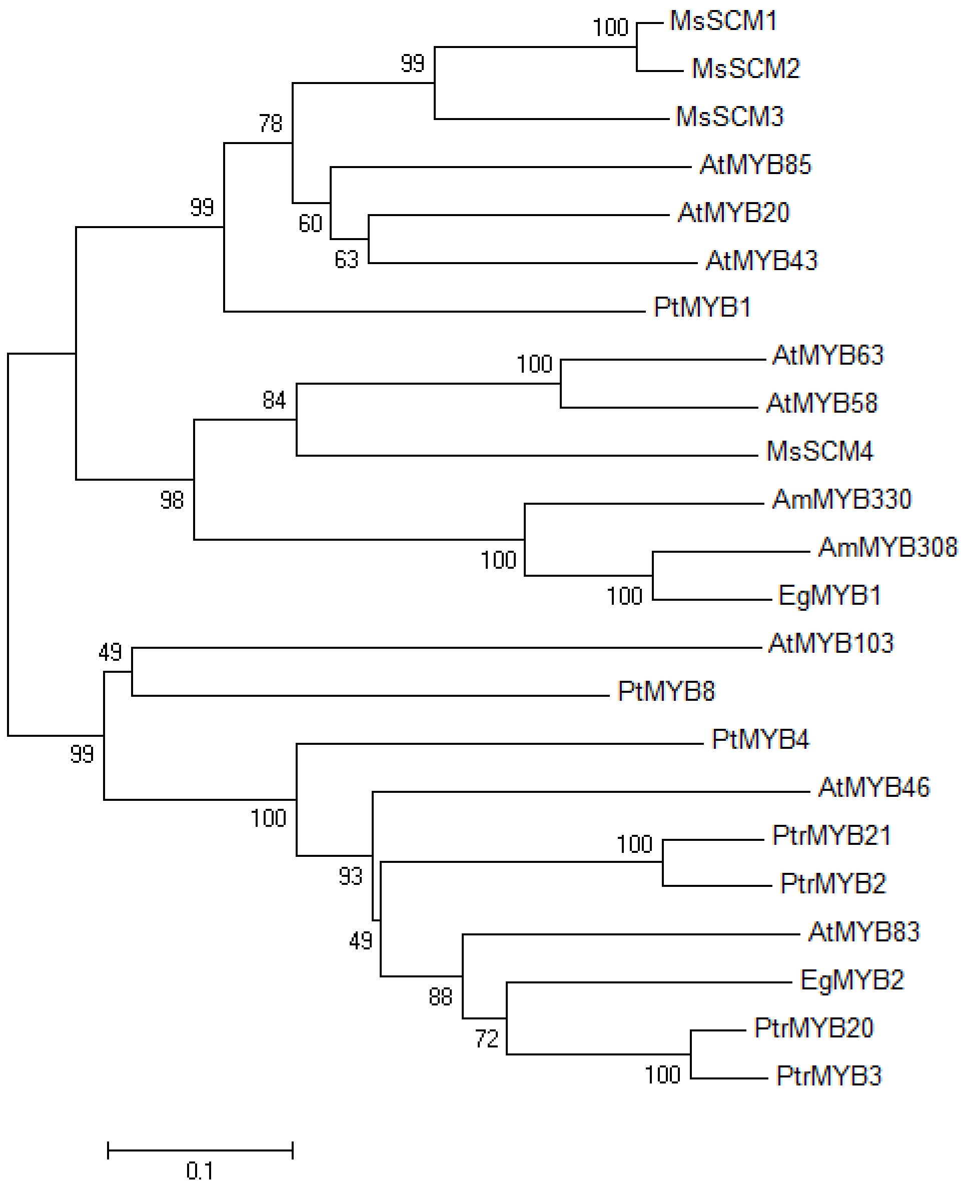


**Supplemental Figure 3.** Phylogenetic tree of MYB transcription factors from angiosperm lineages *Arabidopsis*, *Panicum*, *Populus*, and *Eucalyptus*. Amino acid sequences were aligned with ClustalW and the neighbour-joining phylogenetic tree with 1000 bootstraps was conducted using MEGA6 software (Tamura et al., 2013).

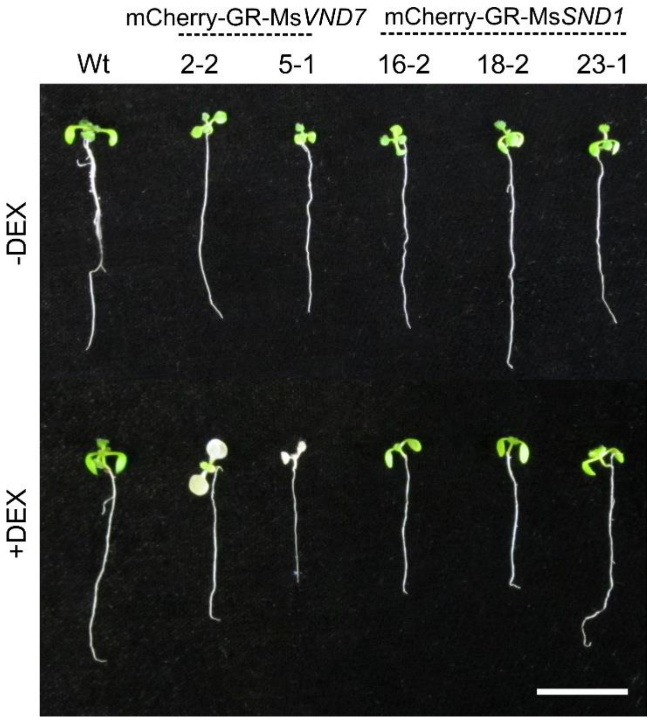


**Supplemental Figure 4.** Induction of mCherry-GR-Ms*VND7* and mCherry-GR-Ms*SND1* Arabidopsis lines with DEX for 3 days leads to chlorosis and cell death.

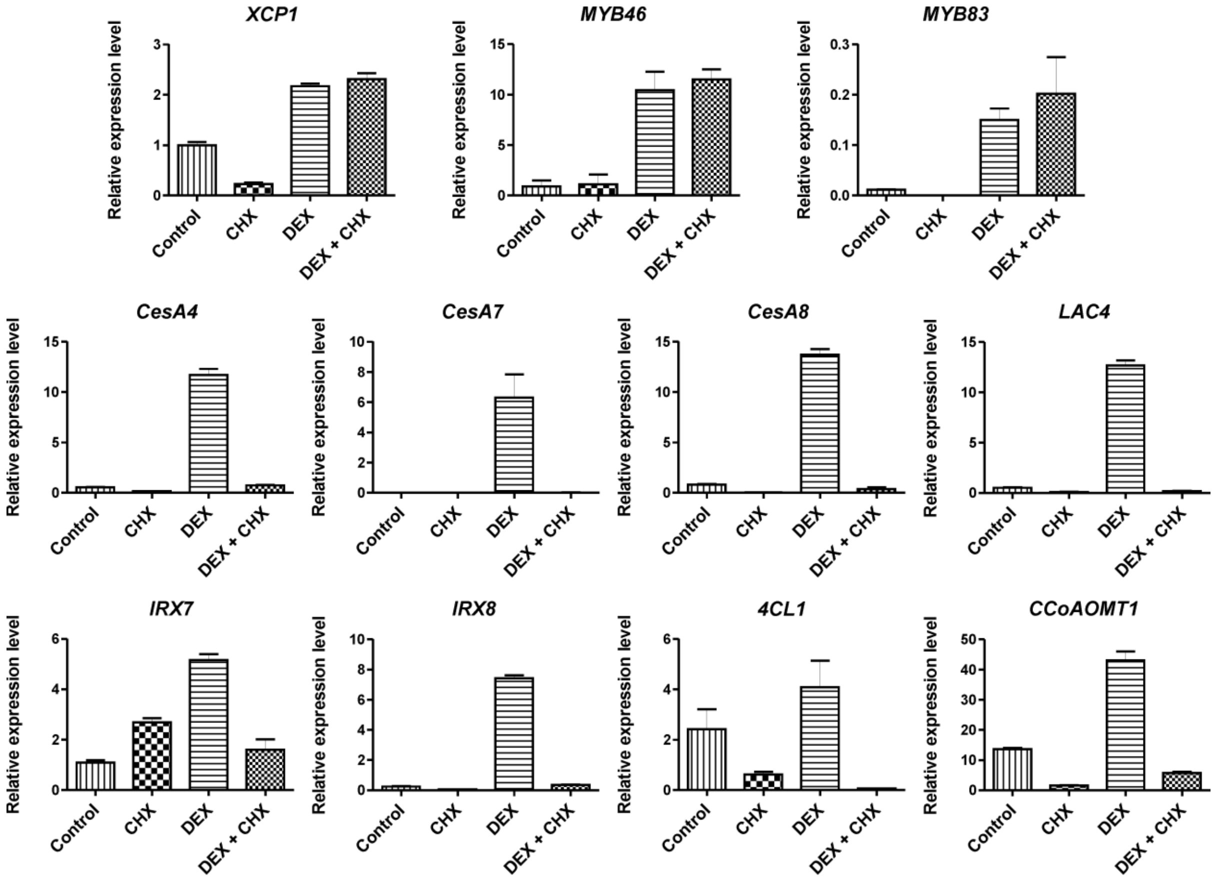


**Supplemental Figure 5.** Expression analysis of candidate genes directly and indirectly targeted by MsSND1 (related to Fig. 7). Ten-days-old heterozygous *Arabidopsis* mCherry-GR-Ms*SND1* seedlings (line 18) were treated with cycloheximide (CHX) and/or dexamethasone (DEX). Gene expression is normalized against clathrin adaptor subunit. Bars depict means ± SE from three technical replicates.
