# Supplementary material for: Regulation of secondary cell wall biosynthesis by a NAC transcription factor from Miscanthus
Source: Plant Direct. 2017 Nov 1;1(5):e00024. doi: 10.1002/pld3.24 (PMC6508536; doi:10.1002/pld3.24)
Supplement: Supplementary file 2 [file PLD3-1-e00024-s002.pdf]

Complementation Constructs	
pPG10	SND1 promoter (At1g32770)
pGGB003	B-Dummy
pPG11/12	MsSND1 or AtSND1 (ORF)
pGGD002	D-Dummy
pGGE009	UBQ10 terminator
pGGF001	pMAS::BastaR::tMAS
pGGZ003	destination vector

Overexpression Constructs	
pGGA004	35S promoter
pGGB003	B-Dummy
pPG11-16/ pGGC087	MsSND1, AtSND1, C-Dummy (control), MsMYB20, MsMYB43, MsMYB63 and MsMYB85
pGGD002	D-Dummy
pGGE001	RBCS terminator
pGGF026	pUBQ::mCherry::tRBCS pNOS::KanR::tNOS
pGGZ003	destination vector

Inducible Construct	
pGGA006	UBQ10 promoter (At4g05320)
pGGB021	mCherry-Glucocorticoid Receptor-linker
pPG11	MsSND1
pGGD002	D-Dummy
pGGE001	RBCS terminator
pGGF005	pUBQ10::HygromycinR::tOCS
pGGZ003	destination vector

Name		Reference	Addgene plasmid #	PCR template	Fwd Oligo	Rev Oligo
pPG10	SND1 promoter (At1g32770)	this publication		Arabidopsis gDNA	aacaGGTCTCAacctCGCGATTATTTGTGCTGAAG	aacaGGTCTCTtgttTCAGCCATTAACGAAGATAGCA
pGGA004	35S promoter	Lampropoulus *et al.*, 2013	48815			
pGGA006	UBQ10 promoter (At4g05320)	Lampropoulus *et al.*, 2013	48816			
pGGB003	B-Dummy	Lampropoulus *et al.*, 2013	48821			
pGGB021	mCherry-Glucocorticoid Receptor-linker	A kind gift from Dr. Miotk (Heidelberg University, Germany)				
pPG11	MsSND1	this publication		Miscanthus cDNA	aacaGGTCTCAggctCAATGAGCATCTCGGTGAACG	aacaGGTCTCTctgaCTAGAAGTTGTTCATCGTCAAGTCC
pPG12	AtSND1 (At1g32770)	this publication		Arabidopsis cDNA	aacaGGTCTCAggctCACGTTAATGGCTGATAATAAGGTCA	aacaGGTCTCTctgaTCATACAGATAAATGAAGAAGTGGGT
pPG13	MsMYB20	this publication		Miscanthus cDNA	aacaGGTCTCAggctCAAGAGAGATGGGCAGGCAG	aacaGGTCTCTctgaCTAGCTAGAATTTTGCTCCGTTTG
pPG14	MsMYB43	this publication		Miscanthus cDNA	aacaGGTCTCAggctCAATGGGGAGACAGCCATGC	aacaGGTCTCTctgaTTAGAAGTTTGATCCATTTGAGGC
pPG15	MsMYB63	this publication		Miscanthus cDNA	aacaGGTCTCAggctCAATGGGGCGAGGGCGAG	aacaGGTCTCTctgaTCATAGCAGAACGGCAGATG
pPG16	MsMYB85	this publication		Miscanthus cDNA	aacaGGTCTCAggctCAATACAGGTTGGGATGGGTCG	aacaGGTCTCTctgaCTAGCTAGCTAGAATTTTGCTCCG
pGGC087	C-Dummy	A kind gift from Dr. Miotk				
pGGD002	D-Dummy	Lampropoulus *et al.*, 2013	48834			
pGGE001	RBCS terminator	Lampropoulus *et al.*, 2013	48839			
pGGE009	UBQ10 terminator	Lampropoulus *et al.*, 2013	48841			
pGGF001	pMAS::BastaR::tMAS	Lampropoulus *et al.*, 2013	48842			
pGGF005	pUBQ10::HygromycinR::tOCS	Lampropoulus *et al.*, 2013	48846			
pGGF026	pUBQ::mCherry::tRBCS pNOS::KanR::tNOS	A kind gift from Dr. Miotk				
pGGZ003	destination vector	Lampropoulus *et al.*, 2013	48869			

Supplementary Table 1. Plasmid constructs and primers for cloning

		Transcript	Forward (5'-3')	Reverse (5'-3')	Product length (bp)
Miscanthus sinensis	Reference	PP2A	GCTAGCTCCTGTCATGGGTC	TCATGTTCGGAACCCTGTCC	89
		Clath	ACAATCAAGGAATTGGGCCG	GCACCGAAAACACTCTTGACT	65
		UBC	CTGAACCAGACAGCCCACTT	CTCTGATATCACCCGACCGC	63
		TIP	ACTGTGGGAGTGATGCTGTG	CTCCCAACACAAAGTGCTGC	80
	Transcription factors	SND1	GACATCCAAGAGAAGTGCCG	CGTCGGGTACTTCTTGTCCT	84
		MYB63	ACATAGCAAGCTTCAGCCCA	CCACCAGGAGTTCAGGTTCC	105
		MYB85	GCAGCCCTACGGAATCGA	CCAGCGGGTCTTGGTCAT	93
		MYB43	AAGGCAGCTTCCTCACAGTC	GCTGGACTGCTCCGATGAAT	132
	Lignin biosynthesis	HCT	GGAGCACTGGATAGGATGGA	AAGTCGGCATCATGGATAGG	161
		CCoAOMT	ACGCCGACAAGGACAACTAC	GTCACGGTAGAAGCGGATGT	155
Arabidopsis thaliana	Reference	Clath	TCGATTGCTTGGTTTGGAAGAT	GCACTTAGCGTGGACTCTGTTTGC	61
	Transcription factors	MYB46	TTCGCTTTCATTCCATCCTCG	CAATCGTGCTGCAATCTGAGA	53
		MYB83	AACGTGGATCCTTCTCTCCTC	AGCCGAGTAGCTATTTGAGACC	91
	Cell wall biosynthesis	CesA4	CTGTGGTTATGAAGAGAAGACTGAA	TGCATTCTAAATCCAGTGAGGA	93
		CesA7	ATGCCACCGATAAGCACATT	TTCCTCAATGCTAACTCCGC	108
		CesA8	GATTCCTACCCAACAGCACAT	ACAGAGCCAAGATGATCAACC	149
		LAC4	GGTAGATATCCAGGTCCCACA	GTTATGTAAGCAGGCCCATCA	149
		IRX7/FRA8	AAAACATCAGTGGACGCTTCT	TTGCCGTTGGAGATAAAACCT	92
		IRX8	GGCTTGGAGGAGGACTAACAT	GTTTGGACATGACCGTGGAAA	132
		4Cl1	CTAATGCCAAACTCGGTCAGG	AGCTCCTGACTTAACCGGAAA	104
		CCoAOMT1	CTCACAAGATCGACTTCAGGG	ACGCTTGTGGTAGTTGATGTAG	137
	Cell death	XCP1	TCCACAAAGAAGATGATTACCCTTA	TCACACGTTCCACATCCTCTT	78

Supplementary Table 2. qPCR primers for Arabidopsis and Miscanthus

TF	Accession-No.	Sequence
Ms*SND1*	KY930620	>MsSND1_CDS<br>ATGAGCATCTCGGTGAACGGGCAGTCGGTGGTACCGCCGGGGTTCCGGTTCCACCCGACGGAGGAGGAGCTGCTGACCTACTACCT<br>GAAGAAGAAGGTGGCGTCGGAGCGCATCGACCTGGACGTCATCCGCGACGTCGACCTCAACAAGCTCGAGCCATGGGACATCCAAG<br>AGAAGTGCCGCATCGGTTCTGGCCCCCAGAACGACTGGTACTTCTTCAGCCACAAGGACAAGAAGTACCCGACGGGGACGCGCACG<br>AACCGCGCCACCGCCGCGGGGTTCTGGAAGGCCACCGGCCGCGACAAGGCCATCTACGCCTCGGGCGCCCGCCGCATCGGCATGC<br>GCAAGACGCTCGTCTTCTACAAGGGCCGCGCACCGCACGGGCAGAAGTCCGACTGGATCATGCACGAGTACCGCCTCGAGGCGGC<br>GCTCGACGCCGCCGCCGGTAGTGCCGCGCACCACCCCGCCGCCGGTGCCGCCGCTGATCACCCCTACTACACCTCGTCGCCGCCT<br>GCTCTTCCTACCGCAATCCGTGGCGCAGCGGCAGAGCAAGCGGCGCAGGAGCAGGAAGGGTGGGTGATCTGCAGGGTGTTCAAGA<br>AGAAGAACCTCGTGCACCACGGCCAGAGCAGCGGCGCCGGCGTGACAGCAGCAGGAAACCACGCGGCGTCCAAGATGGCGGCCGC<br>GGCGGCGCCCATGGACAGCAGCCCAAGCCACTGCTCGTCGGTGACCGTCAGCGACTACTCCAACAAGCAGCAGGCGCAGGCGATG<br>CTGCAGCACTCGGCCAGCGACGACGCGCTCGACCACATCCTGCAGTACATGGGCGGCGGCGGCAAGCAGCCGGACACCAAGCCGG<br>CGCTGCTGGACCACCACCACCACCACGTTGCTGCAGCTACTACCACGACCGCCGCTTGTCCTGCCGGCGTCGGCGGCCTCTACGGG<br>AAGTTCATGAAGCTCCCGCCCCTCGAGCACGCCGGCGCCTGCGGGCTGCTGCCGAGCCCACCGGGGGCGTGCGAGTACGGCGCC<br>GCTGACGCATCAGAGATCGCCGACTGGGACGCGCTGGACCGGCTCGCCGCGTACGAGCTCAACGGCCTCTCCGACGCGTCCAAGA<br>ACATGTCCGCCTTCTTCGACGTCGAGCATGCTAGCGCCGCCGCCGCCTTCTCCTCCTCGTCGTCCGCGCACGTTTCCGCCGCCGTC<br>GACGGCGACCTGTGGAGCCTGGCGAGGTCGGTGTCGGCGTTGCACGCGGACTTGACGATGAACAACTTCTAG
		>MsSND1_AA<br>MSISVNGQSVVPPGFRFHPTEEELLTYYLKKKVASERIDLDVIRDVDLNKLEPWDIQEKCRIGSGPQNDWYFFSHKDKKYPTGTRTNRATAA<br>GFWKATGRDKAIYASGARRIGMRKTLVFYKGRAPHGQKSDWIMHEYRLEAALDAAAGSAAHHPAAGAAADHPYYTSSPPALPTAIRGAAAE<br>QAAQEQEGWVICRVFKKKNLVHHGQSSGAGVTAAGNHAASKMAAAAAPMDSSPSHCSSVTVSDYSNKQQAQAMLQHSASDDALDHILQY<br>MGGGGKQPDTKPALLDHHHHHVAAATTTTAACPAGVGGLYGKFMKLPPLEHAGACGLLPSPPGACEYGAADASEIADWDALDRLAAYELN<br>GLSDASKNMSAFFDVEHASAAAAFSSSSSAHVSAAVDGDLWSLARSVSALHADLTMNNF
Ms*SCM1*	KY930621	>MsSCM1_CDS<br>ATGGGCAGGCAGCCGTGCTGCGACAAGCTGGGGGTGAAGCGGGGGCCGTGGACGGCGGAGGAGGACCGCAAGCTCATCAACTTCA<br>TCCTGACCAACGGCCATTGCTGCTGGCGCGCGGTGCCCAAGCTCGCCGGCCTGCTGCGCTGCGGCAAGAGCTGCCGCCTGCGCTG<br>GACCAACTACCTCCGCCCGGACCTCAAGCGCGGGCTCCTCACGGACGCCGAGGAGCAGGTCGTCATCGACCTCCACGCCAAGCTCG<br>GCAACAGATGGTCGAAGATTGCTGCCAAGCTACCGGGCAGGACTGACAACGAGATCAAGAACCACTGGAACACGCACATCAAGAAGA<br>AGCTGATCAAGATGGGCATCGATCCAGTCACGCACGAGGCCCCCGACCGGAAGACAACCAGCAGCAGCCCGGCTACAACCTCGCAG<br>TCAACCAAGTTCGACGAGGCGAACAAGCAGCAGAGCCCACAGAACGACGACGTCGTCGCAATGAGGGACGTGCCGCCCGATGGTTG<br>CAGCCCGACGGAATCGAGCACGAACACCGTGAGCACCGGCGGAAGCAGCAGCAGTGGCGGTGGCCGCCATGACCCAGATCCGCTG<br>GTGAAGTGGCTCTTGGAAGAGGAGCCTCCCACCGGCAACGAACCGTGGCTCAACTTCACTGGCAGTGTCGATGTGGACGAGTTCAG<br>CAGCATTTCCGCCGGTCCGGAGTTGTTGCCGTGGGATGGCGCGACCGACTGGCTGCTCGACTACCAAGATTATGGATTGGGGGACT<br>CGGCGACCTTGGTCGATGGCTACATGGTCAACAACAGCTCAAACGGAGCAAAATTCTAG
		MsSCM1_AA<br>MGRQPCCDKLGVKRGPWTAEEDRKLINFILTNGHCCWRAVPKLAGLLRCGKSCRLRWTNYLRPDLKRGLLTDAEEQVVIDLHAKLGNRWS<br>KIAAKLPGRTDNEIKNHWNTHIKKKLIKMGIDPVTHEAPDRKTTSSSPATTSQSTKFDEANKQQSPQNDDVVAMRDVPPDGCSPTESSTNTV<br>STGGSSSSGGGRHDPDPLVKWLLEEEPPTGNEPWLNFTGSVDVDEFSSISAGPELLPWDGATDWLLDYQDYGLGDSATLVDGYMVNNSS<br>NGAKF
Ms*SCM2*	MF996502	> MsSCM2_CDS<br>ATACAGGTTGGGATGGGTCGGCAGCCGTGCTGCGACAAGCTGGGGGTGAAGCGGGGGCCGTGGACGGCGGAGGAGGACCGCAAG<br>CTCATCAACTTCATCCTGACCAACGGCCATTGCTGCTGGCGCGCGGTGCCCAAGCTCGCGGGCCTGCTGCGCTGCGGCAAGAGCTG<br>CCGCCTGCGCTGGACTAACTACCTCCGCCCGGACCTCAAGCGCGGGCTCCTCACGGACGCCGAGGAGCAGGTCGTCATCGACGTC

		CACGCCAAGCTCGGCAACAGATGGTCGAAGATTGCTGCCAAGCTACCGGGCAGGACTGACAACGAGATCAAGAACCACTGGAACAC<br>GCACATCAAGAAGAAGCTGATCAAGATGGGCATCGATCCAGCCACGCACGAGCCCCTCGACCGGAAGACAACCAGCAGCAGCCCGG<br>CTACAACCTCGCAGTCAACCAAGTTCGACGAGGCGACCAAGCAGCAGAGCCCGCAGAACGACGACGTCGTCGCGATGAGGGACGTT<br>CCGCCCGATGGTTGCAGCCCTACGGAATCGAGCATGAACACCGTGAGCACCGGCGGAAGCAGCAGCAGCAGCAGCAGTGGCAGCG<br>GCCATGACCAAGACCCGCTGGTGAAGTGGCTCATGGAAGAGGAGCCTCCCACCGGCGACGAGCCGTGGCTCAACTTCACTGGCAGT<br>GTCGATGTGGATGAGTTCAGCAGCATTGCCGCCGGTCTGGAGTTGTTGCCGTGGGATGGCGCGACCGACTGGCTGCTCGACTACCA<br>AGATTTTGGATTGGGGGACTCGGCGACCTTGGTCGATGGCTACATGGTCAACAACAGCTCAAACGGAGCAAAATTCTAG
		> MsSCM2_AA<br>MGRQPCCDKLGVKRGPWTAEEDRKLINFILTNGHCCWRAVPKLAGLLRCGKSCRLRWTNYLRPDLKRGLLTDAEEQVVIDVHAKLGNRWS<br>KIAAKLPGRTDNEIKNHWNTHIKKKLIKMGIDPATHEPLDRKTTSSSPATTSQSTKFDEATKQQSPQNDDVVAMRDVPPDGCSPTESSMNTV<br>STGGSSSSSSSGSGHDQDPLVKWLMEEEPPTGDEPWLNFTGSVDVDEFSSIAAGLELLPWDGATDWLLDYQDFGLGDSATLVDGYMVNN<br>SSNGAKF
Ms*SCM3*	KY930622	> MsSCM3_CDS<br>ATGGGGAGACAGCCATGCTGTGACAAGCAGGGGGTGAAGCGAGGGCCTTGGACAGCGGAGGAGGACAAGAAGCTCATTAGCTTCAT<br>CCTGACACATGGTCGATGCTGCTGGCGGGCAGTGCCTAAACTGGCAGGGCTGCTTCGTTGCGGCAAGAGTTGCCGTCTGCGGTGGA<br>CTAACTACCTCCACCCGGACCTCAAGCGTGGCCTTCTCAGCACTGCCGAGGAGCAGCTCATCATTGACCTCCATGCCAAGCTCGGAA<br>ATAGATGGTCCAAGATTGCTGCCAAGTTACCTGGGAGAACAGACAATGAGATCAAGAACCACTGGAACACGCACATCAAGAAGAAGCT<br>CATCAAGATGGGCATCAACCCGGCCACACACCAGCCTCTAGCCAATTCCAAGGCAGCTTCCTCACAGTCCACAGTTACAGATGAATCT<br>GCTAAATCCAGCGACTTCAGAGAGGAGCTGAGCTTGAAGGATGACAGCCATAGGGAGGTTCCGCTGTCCACTGATTCATCGGAGCAG<br>TCCAGCTGGCCAGAGTCAGGCACCAACGGCTGTGATCAAGACCCTGAACTGCTGGTGAATTGGCTGTCAGAGACAGACCTGTCGATG<br>GATGAGCCATGGCTGAATTTTATGAGCAGCAACGATGAACTTGGCAATGTCCAGGGGATGTTGCCATGGGATGGAACAACAGACTGG<br>CTGCTGGACTACCAAGACTTTGGCATGTGCAGCTCGAACTCAGTCGACAATTCAATGTTCCACGCCTCAAATGGATCAAACTTCTAA
		> MsSCM3_AA<br>MGRQPCCDKQGVKRGPWTAEEDKKLISFILTHGRCCWRAVPKLAGLLRCGKSCRLRWTNYLHPDLKRGLLSTAEEQLIIDLHAKLGNRWSK<br>IAAKLPGRTDNEIKNHWNTHIKKKLIKMGINPATHQPLANSKAASSQSTVTDESAKSSDFREELSLKDDSHREVPLSTDSSEQSSWPESGTN<br>GCDQDPELLVNWLSETDLSMDEPWLNFMSSNDELGNVQGMLPWDGTTDWLLDYQDFGMCSSNSVDNSMFHASNGSNF
Ms*SCM4*	MF996501	> MsSCM4_CDS<br>ATGGGGCGAGGGCGAGCGCCGTGCTGCGCCAAGGTGGGGCTCAACAGGGGCTCCTGGACGCCGCAGGAGGACATGCGTCTCATC<br>GCCTACATCCAGAAGCACGGCCACACCAACTGGCGCGCGCTGCCCAAGCAAGCAGGTTTGCTGCGGTGTGGGAAGAGCTGCAGGCT<br>GCGGTGGATCAACTACCTGCGGCCGGACCTCAAGCGCGGCAACTTCACCGCCGAGGAGGAGGAGACCATCATAAGGCTGCACGGCT<br>TGCTCGGGAACAAATGGTCTAAGATCGCTGCCTGCCTGCCGGGGAGGACGGACAACGAGATCAAGAACGTGTGGAACACGCACCTG<br>AAGAAGAAGGTGGCGCCGCGGGAGAAGCAGAAGGCTGGCGCGGCGGACAAGAACGACGGCGCGGCGAGCGGCGACGCCGGCAC<br>GCTCGCAACTGCCGCCTCGTCCTCGGCGTCCTCCTCGACGACGACCCACAACAGTTCCGGGGGCAGCGACAACTCCGGCGACCAGT<br>GCGGCACGAGCAGGGAGCCCGACGACGCGATCGACGTATCCCTCCTCGGACTAGAAGACATTGATGTCTCGGACATGCTGGTGGAC<br>GCGCCCCCGGCGGCGCAGTCATGCCAGGCGCCAATGCTGTCGCCGTGCTCGTGGTCCTCCCTGACGACGTGCGTCGGCGGCGTGG<br>AGGAGCTGATCGAGATCGAGCCGGAGATCTGGAGCCTCATCGACGGCGAGAGCGCTGGCGCGGCGGACGCCTCGGGCGTGGATGC<br>AACAGCGCCATGTACGGGCACGACCGCCGTCAGCAGCACCAGCGAAGCAGAGGAAGCGGCGAACGATTGGTGGTTGGAGAATTTGG<br>AGAAGGAGCTGGGCCTGTGGGGCCCAGCGGAGGATACTCAGGCCCACCCTGACCTACTGGACCACATAGCAAGCTTCAGCCCACTC<br>GGCGCCTTGGAGTTGGAGAGGGACCCAGTCTCCACCTACTTCCAGACCGCGCCCGCCGTTGCGGAACCTGAACTCCTGGTGGTGGA<br>CGAACCATCTGCCGTTCTGCTATGA
		> MsSCM4_AA<br>MGRGRAPCCAKVGLNRGSWTPQEDMRLIAYIQKHGHTNWRALPKQAGLLRCGKSCRLRWINYLRPDLKRGNFTAEEEETIIRLHGLLGNK<br>WSKIAACLPGRTDNEIKNVWNTHLKKKVAPREKQKAGAADKNDGAASGDAGTLATAASSSASSSTTTHNSSGGSDNSGDQCGTSREPDD

		AIDVSLLGLEDIDVSDMLVDAPPAAQSCQAPMLSPCSWSSLTTCVGGVEELIEIEPEIWSLIDGESAGAADASGVDATAPCTGTTAVSSTSEA EEAANDWWLENLEKELGLWGPAEDTQAHPDLLDHIASFSPLGALELERDPVSTYFQTAPAVAEPELLVVDEPSAVLL

Supplementary Table 3. Nucleotide and amino acid sequences of Miscanthus transcription factors identified in this study
